# Sulfur and nitrogen co-doped carbon quantum dots as fluorescent probes for the determination of some pharmaceutically-important nitro compounds

**DOI:** 10.1038/s41598-023-32494-8

**Published:** 2023-04-04

**Authors:** Galal Magdy, Shaimaa Ebrahim, Fathalla Belal, Ramadan A. El-Domany, Ahmed M. Abdel-Megied

**Affiliations:** 1 Pharmaceutical Analytical Chemistry Department, Faculty of Pharmacy, Kafrelsheikh University, P.O. Box 33511, Kafrelsheikh, Egypt; 2 Pharmaceutical Analytical Chemistry Department, Faculty of Pharmacy, Mansoura University, P.O. Box 35516, Mansoura, Egypt; 3 Microbiology and Immunology Department, Faculty of Pharmacy, Kafrelsheikh University, P.O. Box 33511, Kafrelsheikh, Egypt; 4 Department of Pharmaceutical Sciences, School of Pharmacy, Notre Dame of Maryland University, Baltimore, MD 21210 USA

**Keywords:** Analytical chemistry, Fluorescent probes

## Abstract

In this study, highly fluorescent sulfur and nitrogen co-doped carbon quantum dots (SN-CQDs) were synthesized by a simple one-pot hydrothermal method using thiosemicarbazide and citric acid as starting materials. Various spectroscopic and microscopic techniques were applied to characterize the prepared SN-CQDs. The synthesized SN-CQDs’ maximum fluorescence emission was obtained at 430 nm after excitation at 360 nm. Rifampicin (RFP), tinidazole (TNZ), ornidazole (ONZ), and metronidazole (MNZ) all quantitatively and selectively quenched the SN-CQDs’ native fluorescence, which was the base-for their-spectrofluorimetric estimation without the need for any tedious pre-treatment steps or high-cost instrumentation. SN-CQDs demonstrated a “turn-off” fluorescence response to RFP, TNZ, ONZ, and MNZ over the ranges of 1.0–30.0, 10.0–200.0, 6.0–200.0, and 5.0–100.0 μM with detection limits of 0.31, 1.76, 0.57, and 0.75 μM and quantitation limits of 0.93, 5.32, 1.74, and 2.28 μM respectively. The suggested method was successfully used to determine the investigated drugs in their commercial dosage forms. The method was further extended to their determination in spiked human plasma samples, with satisfactory mean % recoveries (99.44–100.29) and low % RSD values (< 4.52). The mechanism of fluorescence quenching was studied and discussed. The suggested method was validated in accordance with ICH recommendations.

## Introduction

**Figure 1:**
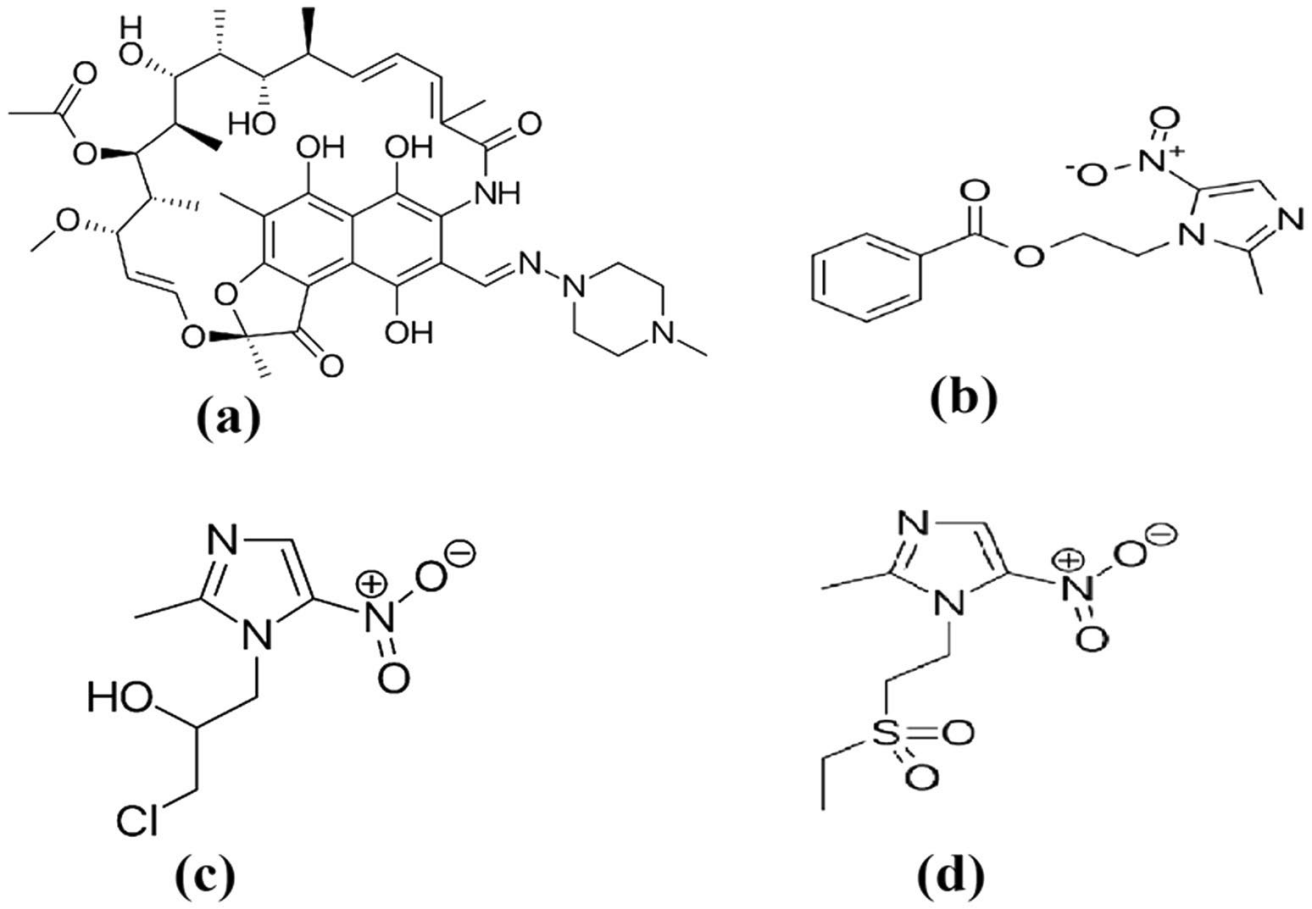
Chemical structure of rifampicin (**a**), metronidazole (**b**), ornidazole (**c**), and tinidazole (**d**).

Rifampicin (RFP) is a semi-synthetic macrocyclic compound with antibiotic activity produced by *Streptomyces mediterranei* and is used to treat tuberculosis, leprosy, and other infectious disorders. It is one of the first-line antituberculous drugs^1^. Chemically, RMP is 3-[[4-methyl-1-piperazinyl)-imino]-methyl]-rifamycin SV^2^, as shown in Fig. 1a.

Literature survey revealed number of published papers for RFP determination in biological fluids and pharmaceutical formulations, including spectrophotometry, spectrofluorimetry, high performance liquid chromatography (HPLC), thin layer chromatography (TLC), electrochemistry, and-capillary electrophoresis, which are summarized in a recent review reported by Kotadiya et al.^3^.

Metronidazole benzoate (MNZ), tinidazole (TNZ), and ornidazole (ONZ) belong to nitroimidazole antimicrobial agents^4^. Because of their exceptional efficacy and comparatively low toxicity, nitroimidazoles have become one of the most well-known antimicrobial agents. This group's members have a wide spectrum of therapeutic actions against anaerobic pathogens, including both gram-positive and gram-negative bacteria, as-well-as protozoans^5^.

Chemically, MNZ is 2-(2-methyl-5-nitroimidazol-1-yl)ethyl benzoate (Fig. 1b)^6^, while ONZ is 1-chloro-3-(2-methyl-5-nitroimidazol-1-yl)propan-2-ol (Fig. 1c)^7^, and the chemical name of TNZ is 1-(2-ethylsulfonylethyl)-2-methyl-5-nitroimidazole (Fig. 1d)^8^. Several methods for analysis of MNZ, TNZ and/or ONZ in bulk and dosage forms, as-well-as-biological-fluids, have been published. These methods include-spectrophotometry^9–14^, spectrofluorimetry^15–19^, HPLC^20–22^, electrochemistry^23–27^, non-aqueous titration^28^, flow injection analysis^29^, capillary electrophoresis^30–32^, and gas chromatography^33^.

Due to their excellent photo-physical properties, high quantum yields, superior solubility, low toxicity, low-cost, and eco-friendly nature with strong fluorescence emission, carbon-quantum-dots (CQDs)-have piqued the-interest-of researchers in a variety of fields^34–37^. The top-down^38,39^ and bottom-up^40^ approaches-for synthesis-of CQDs have both been reported^41^. Larger carbon structures are divided into smaller ones during the top-down processes used to manufacture CQDs, such as electrochemical oxidation and laser ablation, which resulted in low yields and high costs. The carbonization of organic components under thermal, hydrothermal, and solvothermal conditions is a key component of bottom-up approaches, and they have several advantages over top-down methods, including higher yields, unique photophysical features, low cost, and ease of use^42^. Bio-imaging, food processing, drug delivery, light-emitting diodes, photo-catalysis, photo-detectors, and photodynamic treatment are all important examples of CQD applications^43^. Many of the reported methods for synthesis of CQDs need high equipment costs and complicated procedures, and also produce CQDs with low quantum yields. This study used a simple one-pot hydrothermal technique to make SN-CQDs using citric acid as a carbon source and thiosemicarbazide as a nitrogen and sulfur source^44^.

The introduction of heteroatoms, such as boron (B), sulfur (S), nitrogen (N), and phosphorous (P) into the overall structure-of-CQDs, with or without surface changes, is known as doping. Doping CQDs increases their quantum yields and improves their fluorescence qualities, allowing them to be used in various applications^45–51^. Because nitrogen has an atomic radius similar to that of carbon, and sulfur and carbon have similar electronegativity, SN-CQDs represent one of the most important doped-CQDs^52^.

The inner filter effect (IFE), which was once regarded to be an analytical error has recently started to find its place in the field of analysis, as a significant-quenching-process-involving an energy conversion that is-not-linked to radiation^53^. The complementary overlap of the absorber's UV absorption band and the fluorophore's excitation and/or emission band results in a reduction in fluorescence intensity. Using nanosensors based on IFE, eliminates the need for any direct chemical interaction between the analyte and the sensor, providing benefits such as sensitivity, low cost, and simplicity. For the detection and-determination-of many metal-ions, medicines, and-proteins, the main quenching mechanism was proved to be IFE^54^.

When the developed method was compared to other previous published studies for determining RFP, TNZ, ONZ, and MNZ using fluorescent quantum dots^15,17,18,54–57^, it was found that the synthesized SN-CQDs has higher quantum yield (55%) than those of other reported methods. The suggested synthetic approach in the current study has a number of advantages over the previously reported methods for the synthesis of CQDs, which may require the use of expensive instrumentation, a lot of time, or complicated chemical interactions such as boiling with concentrated sulfuric acid as in carbonizing organics. Actually, these intricate prerequisites will undoubtedly cost a lot, take a lot of time, and consequently reduce the method's greenness. Moreover, the proposed method is simple, cost-effective, and easy compared to previously reported ones, such as TLC, HPLC, capillary electrophoresis, and electrochemistry, which required more stringent experimental conditions and sophisticated instrumentation. A detailed comparison between the proposed method and the previously reported ones was summarized in Table 1.

**Table 1:** Comparison of the proposed method to the previously reported methods for determination of the studied drugs.

Analyte	Method	Matrix	Linearity range	LOD	Refs.
MNZ	Extractional spectrophotometry	Pure and dosage forms	2.50–22.50 µg/mL	5.33 × 10^−2^ µg/mL	^9^
	UV-spectrophotometry	Pure and dosage forms	2.0–16.0 µg/mL	–	^12^
	RP-HPLC	Pure and dosage forms	0.10–0.30 mg/mL	–	^22^
	Flow injection analysis	Pure and dosage forms	2.0–20.0 mg/L	0.7 mg/L	^29^
	Capillary electrophoresis	Porcine muscle samples	5.00–300.0 µg/kg	0.5 µg/kg	^32^
	Spectrofluorimetry (N-CDs were synthesized by a microwave-assisted method)	Real milk samples	0.5–22.0 µM	0.22 µM	^15^
	Spectrofluorimetry (CDs were synthesized by a hydrothermal technique)	Pure and dosage forms	3.3 × 10^−6^—2.4 × 10^−4^ M	1.2 × 10^−7^ M	^18^
ONZ	Extractional spectrophotometry	Pure and dosage forms	7.50–35.0 µg/mL	5.01 × 10^–2^ µg/mL	^9^
	Spectrophotometry	Pure and dosage forms	20.0–300.0 µg/mL	–	^11^
	Spectrophotometry	Pure and dosage forms	5.0–25.0 µg/mL	–	^13^
	RP-HPLC	Pure and dosage forms	0.125–0.375 mg/mL	–	^22^
	Square wave adsorptive stripping voltammetry (SWAdsSV) and Differential pulse adsorptive stripping voltammetry (DPAdsSV)	Pure and dosage forms	8.0 × 10^−7^–1.0 × 10^−5^ M	7.6 × 10^–8^ M for DPAdsSV 3.4 × 10^−8^ M for SWAdsSV	^23^
	Capillary zone electrophoresis	Pure and dosage forms	25.0–250.0 μg/mL	1.80 ± 0.06 μg/mL	^30^
	Capillary electrophoresis	Rabbit blood	1.0–412.0 μg/mL	0.7 μg/mL	^31^
	Spectrofluorimetry (bovine serum albumin quenching)	Pure and dosage forms	0.4–8.0 µg/mL	0.106 μg/mL	^16^
	Spectrofluorimetry (graphene-quantum dot embedded silica molecular imprinted polymer (GQD-SMIP))	Plasma samples	0.75–30.0 μM	0.24 μM	^17^
TNZ	Extractional spectrophotometry	Pure and dosage forms	2.50–30.0 µg/mL	5.16 × 10^−2^ µg/mL	^9^
	Spectrophotometry	Pure and dosage forms	20.0–250.0 µg/mL	–	^11^
	Spectrophotometry	Pure and dosage forms	10.0–80.0 µg/mL	–	^14^
	RP-HPLC	Pure and dosage forms	0.125–0.375 mg/mL	–	^22^
	Capillary electrophoresis	Rabbit blood	1.0–520.0 μg/mL	0.6 μg/mL	^31^
	Spectrofluorimetry (Mn-modified CdSe/CdS quantum dots)	Pure and dosage forms	4.0–400.0 μM	0.4 μM	^55^
RFP	Spectrophotometry	Pure and dosage forms	5.0–50.0 µg/mL	3.50 µg/mL	^3^
	Spectrophotometry (Chelate Formation and Charge Transfer Complexation)	Pure, dosage forms, and biological fluids	− 5.0 to 140.0 µg/mL − 2.0 to 45.0 µg/mL − 5.0 to 120.0 µg/mL − 15.0 to 200.0 µg/mL − 10.0 to 240.0 µg/mL	0.90—3.95 µg/mL	^3^
	RP-HPLC	Human Plasma	40.0–100.0 µg/mL	0.13 µg/mL	^3^
	High-performance Thin-layer chromatography (HPTLC)	Pure and dosage forms	1.0–3.0 µg/µL	0.035 µg/spot	^3^
	Capillary Zone electrophoresis	Pure and dosage forms	30.8–57.2 mg/L	0.34 mg/L	^3^
	Spectrofluorimetry (P and N Co-doped passivated carbon nanodots)	Dosage forms	1.0–100.0 μM	0.06 μM	^54^
MNZ	Spectrofluorimetry (SN-CQDs were synthesized by One-pot Hydrothermal technique)	Pure, dosage forms, and human plasma samples	5.0–100.0 µM	0.75 µM	This method
ONZ			6.0–200.0 µM	0.57 µM	
TNZ			10.0–200.0 µM	1.76 µM	
RFP			1.0–30.0 µM	0.31 µM	

## Experimental

### Materials and reagents

- TNZ (% purity 100.1) and ONZ (% purity 99.68) were kindly supplied by Organopharma (Cairo, Egypt) and Sigma Pharmaceutical Co. (Quesna, Egypt), respectively.
- MNZ (% purity 99.5) and RFP (99.9% purity) were kindly supplied by Hubei Hongyuan Pharmaceutical Technology Co. (Luotian, China) and Novartis Pharma (Cairo, Egypt), respectively.
- Isoniazid (% purity 99.19) was obtained from Tianjin Handewel pharma Co. (China).
- Protozole® coated tablets (Batch No. 210778) labeled to contain 500 mg/tablet, Product of Union Medical Association, Ismaelia, Egypt. Astranida® coated tablets (Batch No. 19052A) labeled to contain 500 mg/tablet, product of PHARMED Company. Flagyl® coated tablets (500 mg MNZ/tablet, Batch No. BEG029), product of Sanofi Aventis, Cairo, Egypt. All preparations were obtained from a local Pharmacy.
- Citric acid (CA), thiosemicarbazide (TSC), dipotassium hydrogen phosphate, potassium dihydrogen phosphate, sodium hydroxide, phosphoric acid, hydrochloric acid (37% w/v), methanol, ethanol, urea, lactose, maize starch, magnesium stearate, talc, boric acid, and glacial acetic acid were obtained from Sigma-Aldrich (St. Louis, MO, USA).
- Analytical grade materials and reagents were used throughout the study and all solutions were prepared with double distilled water. Phosphate buffer (0.05 M, pH 3–9.5) and Britton-Robinson buffer (0.02 M, pH 2–12) were freshly prepared as stated in the United States Pharmacopeia (USP)^58^.
- Mansoura University Hospital (Mansoura, Egypt) provided human plasma samples, which were kept frozen at − 80 °C and gently thawed before use.

### Instrumentation

Agilent Technologies' Cary Eclipse® Fluorescence spectrophotometer with Xenon flash lamp was used (Santa Clara, CA 95051, United States). The instrument voltage was set to 750 V, the smoothing factor was 20, and the slit width was 5 nm for the excitation and emission monochromators. Jenway 3510 pH meter (Jenway-UK) was used for pH measurements. A double beam spectrophotometer was used to perform UV–Vis spectrophotometric measurements (PG Instrument, UK). The membrane filters with pore sizes of 0.45 m were also used (Phenomenex, USA). Thermo Fisher Scientific Fourier Transform Infrared (FT-IR) Spectrometer was used to record FT-IR spectra (Nicolet—iS10, USA). All measurements were obtained between 4000 and 1000 cm^−1^ and recorded as 32 scans at a resolution of 4 cm^−1^. JEM-2100 High Resolution Transmission Electron Microscopy (HRTEM) was used to examine the synthesized SN-CQDs’ morphology. The sample was examined using a Cu-grid coated with carbon (200 mesh) and a 200 kV working voltage (JEOL, Tokyo). Centrifuge, model 2-16P, (Germany), ultrasonic bath, model SS 101H 230 (USA), and vortex mixer, model IVM-300p (Gemmy industrial Corp, Taiwan) were also used.

### Stock solutions

Stock solutions (1.0 mM) of RFP, TNZ, ONZ, and MNZ were separately prepared in methanol. Different working concentrations were obtained by diluting the stock solutions with double distilled water. The stock solutions of TNZ, ONZ and MNZ were stable for at least one week, while that of RFP was stable for more than two weeks when kept at the refrigerator protected from light.

### Synthesis of SN-CQDs

Using CA as a carbon source and TSC as sulfur and nitrogen source, SN-CQDs were prepared using a previously reported one-pot hydrothermal technique^44^. Ultrasonication was used to mix TSC (0.68 g) and CA (0.52 g) with double distilled water (20.0 mL). The mixed solution was then refluxed at 160 °C until fluorescent SN-CQDs formed a dark orange color, then the solution was cooled and kept in-the refrigerator for future use.

### Spectrofluorimetric measurements

100.0 μL aliquots of SN-CQDs were added to 50.0 μM of each ONZ, TNZ, or MNZ and 10.0 μM of RFP solution to optimize factors that affect cited drugs’ fluorescence sensing. Using 360 nm as an excitation wavelength, the emission fluorescence intensities were-measured-at 430 nm. To 100.0 μL of SN-CQDs, serial concentrations of each drug were transferred to a set of 10.0 mL volumetric flasks using 1.0 mL of Britton-Robinson buffer (0.02 M) with pH of 6.0 and 5.1 for RFP and ONZ, respectively. While, 1.0 mL of phosphate buffer (0.05 M, pH = 7.1) with incubation time of 10.0 min was used for MNZ and 0.5-mL of phosphate-buffer (0.05 M, pH = 7.1) was used for TNZ. The resulting fluorescence quenching spectra were recorded at 25 °C. By plotting the decrease in fluorescence intensity against the final concentration of each drug (μM), the calibration curves were created. The corresponding regression equations were also derived.

### Analysis of drugs in dosage forms

A set of laboratory-prepared RFP capsules was made with maintaining the drug’s pharmaceutical concentration. Into a small conical flask, an accurately weighed amount of the powder equivalent to equal to 300.0 mg RFP was transferred, then 40.0 mL of methanol was added. After 15.0 min of sonication, the solution was filtered into a 100.0 mL measuring flask. Few mLs of methanol were used to wash the flask and the-washing was added to the filtrate, then the solution was completed to the volume with methanol. Different aliquots of the filtrate were transferred into 10.0 mL volumetric flasks and completed to the mark with double distilled water to get solutions covering the concentrations range. The corresponding regression equation was used to calculate the nominal contents of capsules.

Ten tablets of each of Astranida**®**, Flagyl**®** and Protozole**®** were separately weighed and crushed. An amount of powder equivalent to 500 mg of each of MNZ, TNZ, and ONZ was transferred separately into a small conical flask, and then the method was continued as described above.

### Procedure for spiked human plasma

Into a series of Falcon tubes (15.0 mL), 1.0 mL aliquots of human plasma were added. Aliquots from stock solutions of RFP, TNZ, ONZ, and MNZ were-spiked-to give final-concentrations-in-the range of 1.0–4.0, 10.0–40.0, 6.0–45.0, and 5.0–35.0 μM, respectively. The solutions were vortex-mixed for 30 s, then 1.0 or 0.5 mL of phosphate-buffer (0.05 M, pH = 7.1) was-added in case of MNZ and TNZ, respectively, while 1.0 mL of Britton-Robinson buffer (0.02 M) with pH of (6.0 and 5.1) was added in case of RFP and ONZ, respectively, followed by 100.0 μL aliquots of SN-CQDs in all cases. Protein precipitation was performed by completing up to 5.0 mL using methanol for ONZ and RFP and acetonitrile for MNZ and TNZ. Centrifugation for 20 min at 6000 rpm was then performed. Supernatant aliquots of one milliliter were measured and filtered by 0.45 μm pore size syringe filters. The drug concentration measurements were carried out as mentioned under “Spectrofluorimetric measurements” Section. A blank plasma sample was measured using the same procedure. To construct the calibration graphs and derive the corresponding regression equations, the-relative fluorescence intensity quenching was plotted against the drug concentrations (μM). The percentage recoveries of studied drugs were determined adopting the corresponding regression equations. All methods were performed in accordance with relevant guidelines and regulations and all experimental protocols were approved by the Committee of Research Ethics in the Faculty of Pharmacy, Kafrelsheikh University, Kafrelsheikh, Egypt.

### Quantum yield measurements

The obtained dots' quantum yield was determined adopting the single point method. The SN-CQDs' fluorescence quantum yield was determined using the following equation^59^:

1 $${\Phi }_{SN-CQDs} = {\Phi }_{QS} \times ({F}_{SN-CQDs}/{F}_{QS}) \times ({A}_{QS}/ {A}_{SN-CQDs}) \times {({\eta }_{SN-CQDs}/{\eta }_{ QS})}^{2}$$

where Φ stands for the quantum yield, F represents the intensity of integrated fluorescence emission, A stands for the absorbance value, and η denotes the solvent's refractive index (double distilled water).

The standard fluorophore used was quinine sulfate (QS), at 350 nm, its quantum yield in 0.1 M H_2_SO_4_ is 0.54. η_NS-CQDs_/η_St_ equals one in aqueous solutions.

### Ethics declarations

All experiments were performed in accordance with relevant guidelines and regulations and this work was approved by the Committee of Research Ethics in the Faculty of Pharmacy, Kafrelsheikh University, Kafrelsheikh, Egypt.

### Informed consent

A waiver for the informed consent for the current study was obtained from the Committee of Research Ethics in the Faculty of Pharmacy, Kafrelsheikh University, Kafrelsheikh, Egypt.

## Results and discussion

A sufficiently sensitive spectrofluorimetric method for determining the investigated drugs in their formulations and biological fluids is needed. SN-CQDs, which are fluorescent nanosensors with strong blue fluorescence intensities that could be quantitatively quenched by studied drugs, could successfully achieve this goal in the current study, providing a rapid, simple, and cost-effective method for their determination.

### Characterization of SN-CQD

**Figure 2:**
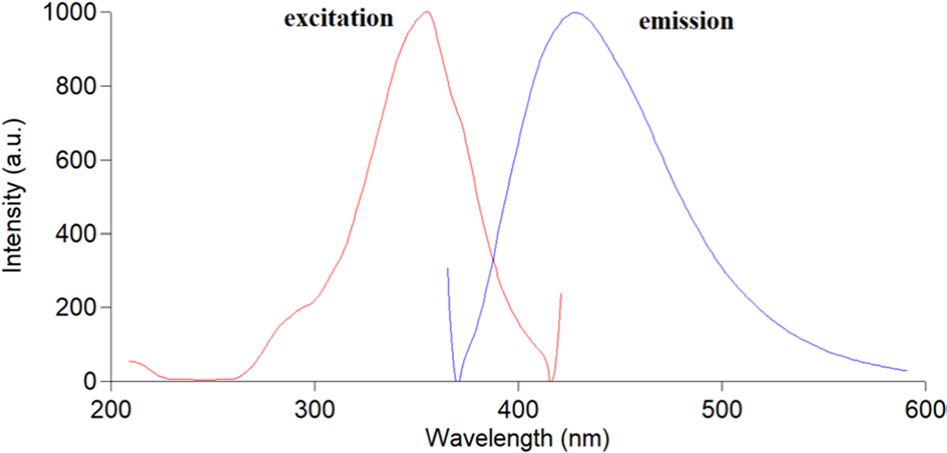
Fluorescence emission spectrum of SN-CQDs at 430 nm after excitation at 360 nm.

**Figure 3:**
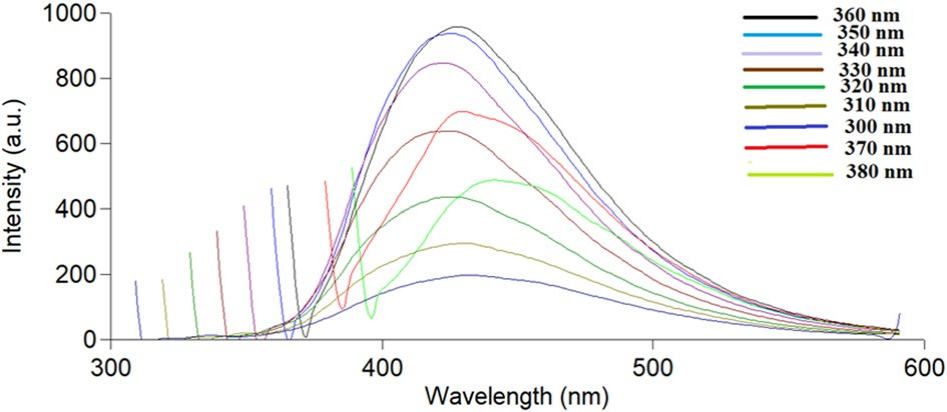
Fluorescence emission spectra of SN-CQDs at different excitation wavelengths (300–380 nm).

A variety of techniques were used to characterize the prepared SN-CQDs. Their optical characteristics were evaluated using fluorescence spectroscopy and UV–Vis absorption. TSC, CA, and SN-CQDs UV–Visible absorption spectra were recorded (Fig. S1) and the surface states that trap excited-state energy are what caused SN-CQDs to have an obvious peak at about 320 nm^44^. When the excitation wavelength was set at 360 nm, the optimum SN-CQDs’ fluorescence intensity could be measured at 430 nm, as shown in Fig. 2. Excitation-dependent photoluminescence was observed, as shown in Fig. 3, where the dots produced different emission peaks when the excitation wavelength was changed from 300 to 380 nm. In addition, SN-CQDs produced strong blue fluorescence when exposed to UV light, and the solution remained stable for about two weeks without alteration. The amino, carbonyl, and hydroxyl groups were found to be responsible for the SN-CQDs' excellent hydrophilicity and high fluorescence^41,52^.

**Figure 4:**
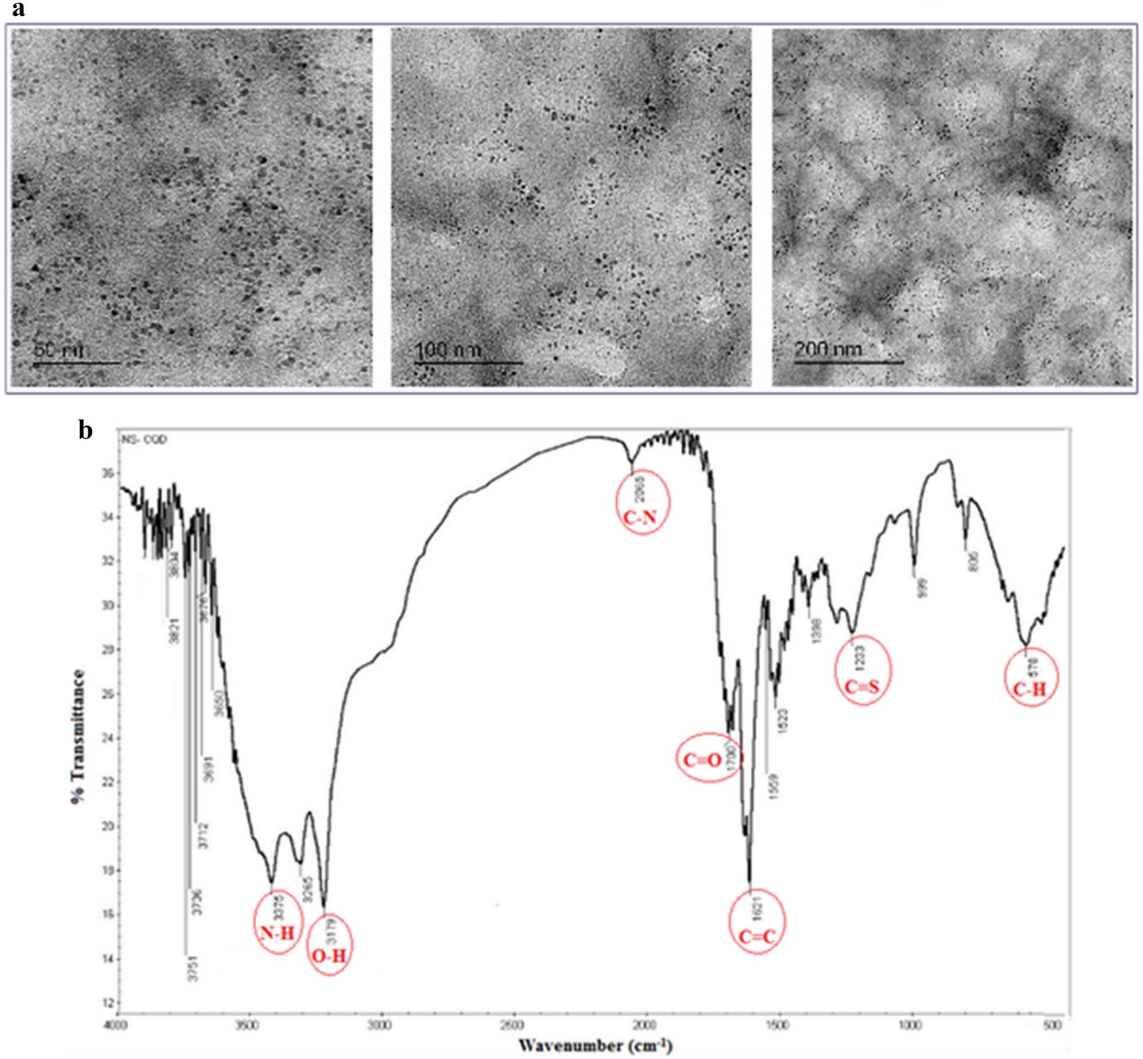
(**A**) The typical HRTEM images of the SN-CQDs, (**B**) FT-IR spectra of SN-CQDs.

HRTEM was also used to study the SN-CQDs' size and shape. At 200 kV voltage, the sample was examined on carbon coated Cu-grid (200 mesh). As shown in Fig. 4A, the range of the particle size distribution was 5–10 nm, and the dots were well-dispersed with spherical forms and isolated from one another without noticeable aggregation.

FT-IR was also used to investigate the SN-CQDs’ surface functional groups (Fig. 4B). The N–H/O–H stretching vibration is characterized by a large peak in the region of 3500–3100 cm^−1^. The stretching vibration of C–N is responsible for the peak at 2065 cm^−1^. The carbonyl group C=O produces a strong peak at 1700 cm^−1^. Furthermore, C=C and C=S create the typical peaks at 1621 and 1233 cm^-1^, respectively, while the C–H bond is demonstrated by a bending peak at 578 cm^−1^^44^.

### Mechanism of fluorescence quenching

**Figure 5:**
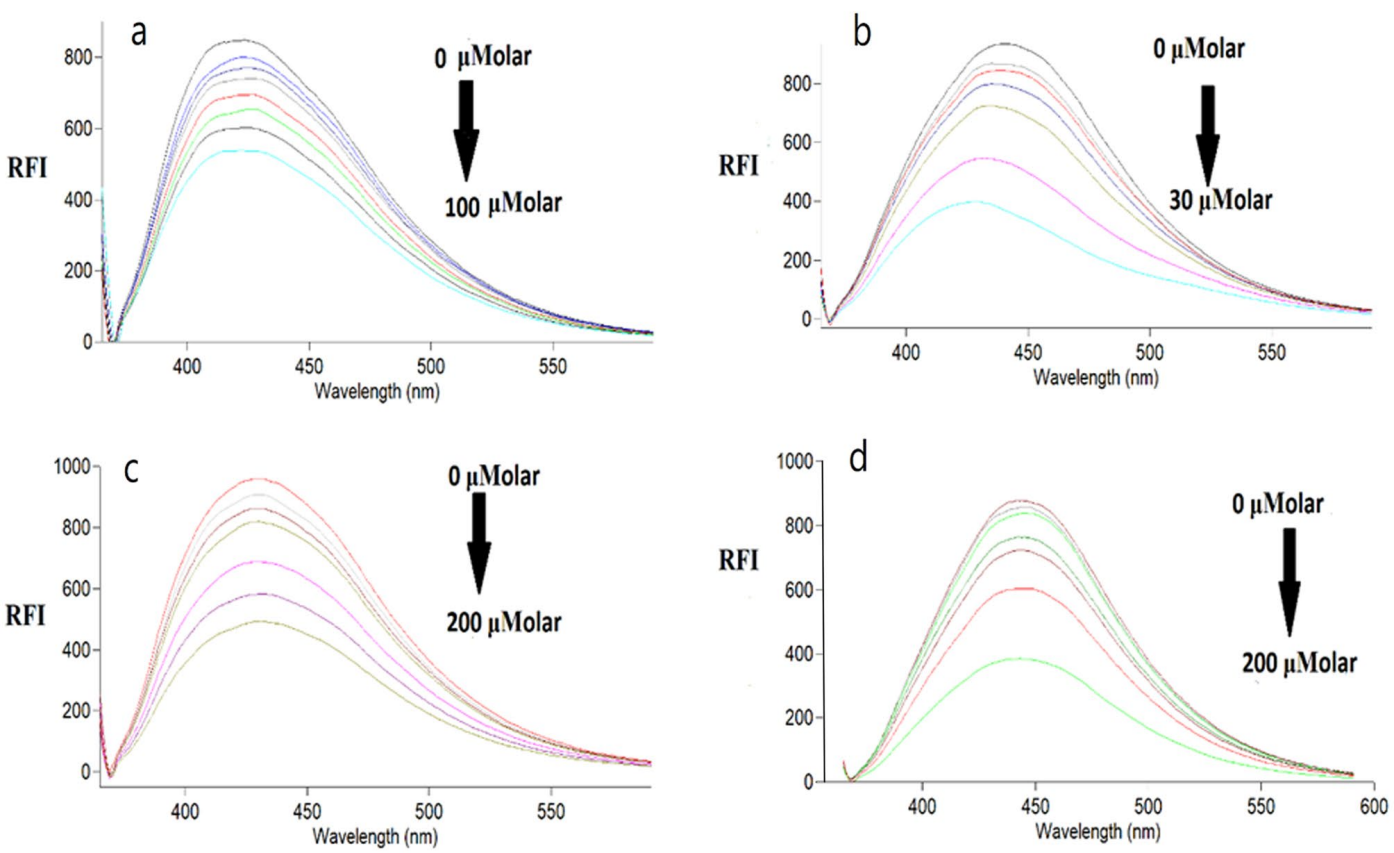
Fluorescence emission spectra of SN-CQDs in aqueous solution upon addition of various concentrations of (**a**) MNZ (from top to bottom: 0, 5.0, 10.0, 25.0, 40.0, 50.0, 75.0, 100.0 μM), (**b**) RFP(from top to bottom: 0, 1.0, 2.0, 5.0, 10.0 20.0, 30.0 μM), (**c**) TNZ (from top to bottom: 0, 10.0, 25.0, 50.0, 100.0, 150.0, 200.0 μM), and (**d**) ONZ (from top to bottom: 0, 6.0, 12.0, 25.0, 50.0, 100.0, 200.0 μM).

Upon addition of RFP, TNZ, ONZ, and MNZ to the produced SN-CQDs, the native fluorescence was quantitatively quenched. The fluorescence emission spectra of the prepared dots with increasing concentrations of the studied drugs are shown in Fig. 5. Generally, fluorescence is quenched by variety of mechanisms, like dynamic quenching, static quenching, and the inner filter effect (IFE)^60^.

**Figure 6:**
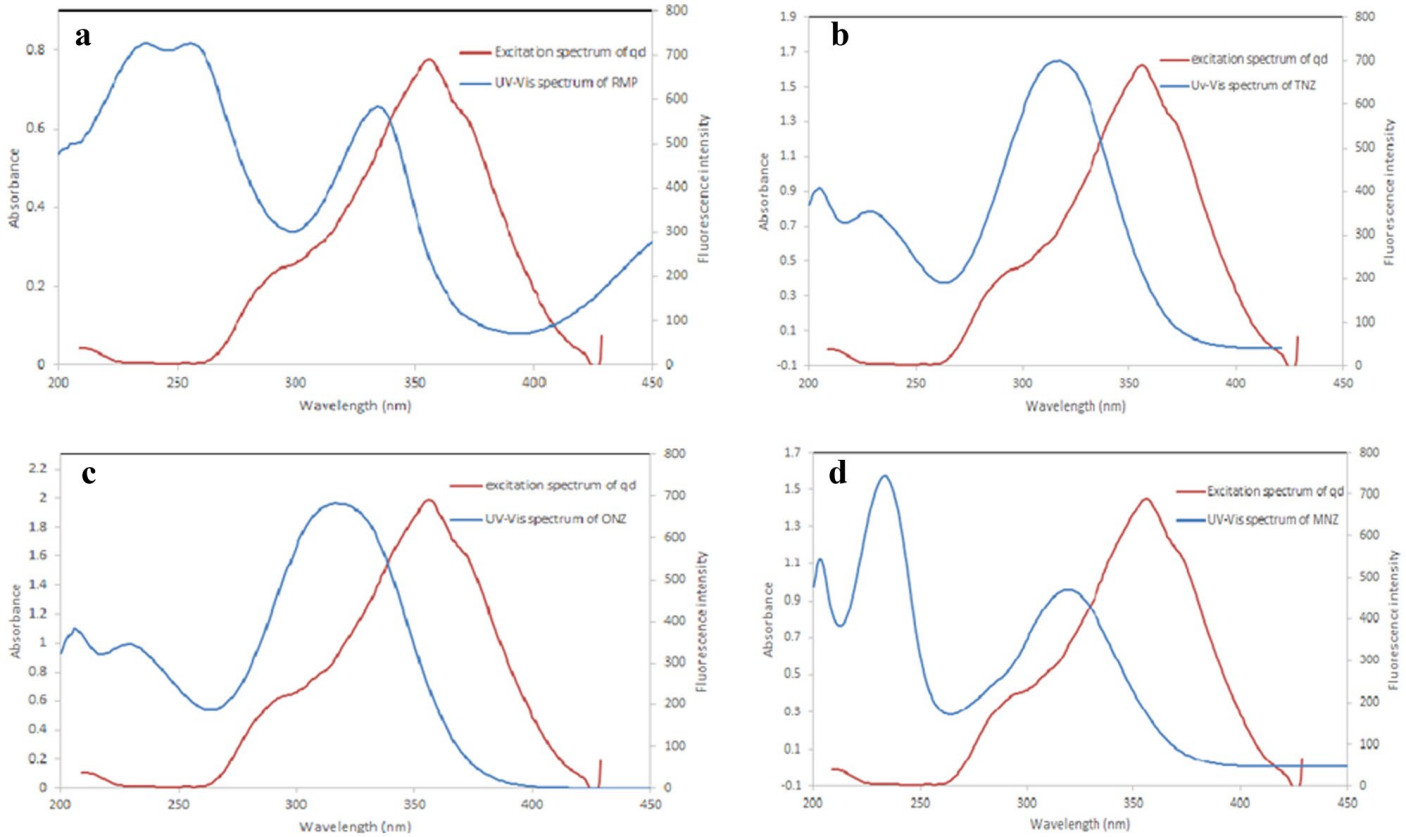
A Co-plot showing the overlap between UV–Vis absorption spectra of RFP (**a**), TNZ (**b**), ONZ (**c**), and MNZ (**d**) and the fluorescence excitation spectrum of the SN-CQDs.

In this study, because of the overlapping between the cited drugs’ UV-absorbance spectra and the SN-CQDs’ excitation spectrum (Fig. 6), IFE is possible to be the quenching mechanism. Upon increasing the concentrations of the quenchers (RFP, TNZ, ONZ, and MNZ), the SN-CQDs fluorescence intensity was corrected for probable IFE using Eq. (2)^61^:

2 $${F}_{corr.}={F}_{obs.} x {10}^{(Aex+Aem)/2}$$

where F_corr._ and F_obs._ refer to the corrected fluorescence intensities after excluding the IFE from F_obs._ and the observed fluorescence, respectively. A_ex_ and A_em_ are the quenchers’ absorbance at the excitation and emission wavelength of SN-CQDs, respectively.

For both corrected and observed fluorescence, the suppressed efficiency (%E) was calculated using Eq. (3)^61^:

3 $$\%E=[1-(F/{F}_{0})] \times 100$$

**Figure 7:**
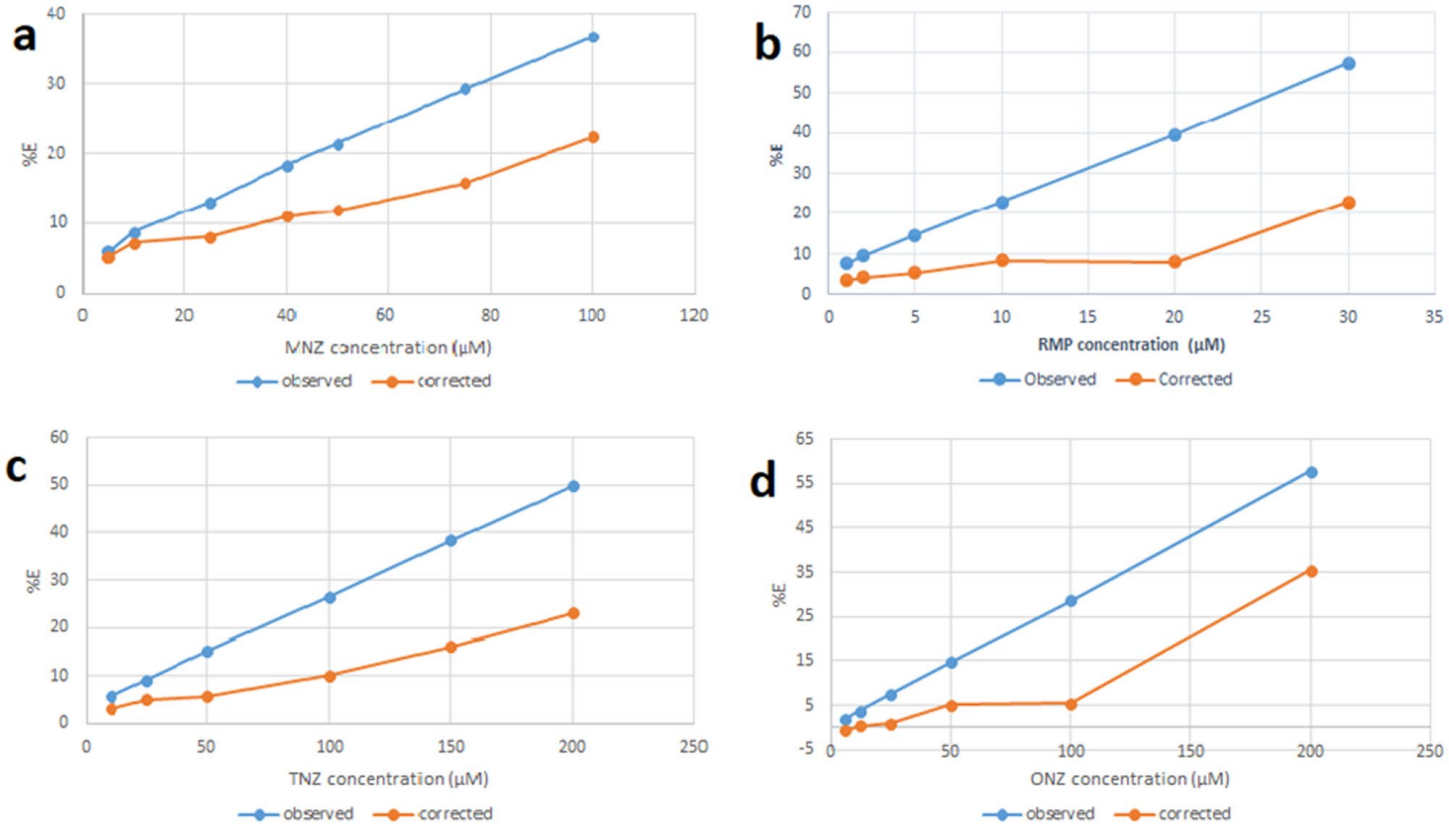
Suppressed efficiency (%E) of observed and corrected fluorescence of SN-CQDs after addition of different concentrations of MNZ (**a**), RFP (**b**), TNZ (**c**), and ONZ (**d**).

The % E plot of corrected and observed SN-CQDs fluorescence intensity versus RFP, TNZ, ONZ, and MNZ concentrations in μM (Fig. 7) demonstrated that, IFE plays a significant role in the quenching of SN-CQDs native fluorescence, this because the studied drugs showed significant absorbance at 360 nm.

In addition to IFE, other mechanisms might occur. Stern–Volmer Eq. (4)^61^ was used to determine the other possible mechanisms that could be responsible for the quenching of SN-CQDs’ native fluorescence.

4 $${F}_{0}/F=1+{K}_{sv}[Q]=1+{K}_{q}\tau 0[Q]$$

where F_0_ and F represent the fluorescence intensities in the absence and presence of quencher, respectively, k_q_ denotes the bimolecular quenching rate constant, K_sv_ represents the Stern–Volmer quenching constant. The average lifetime (10^−8^ s) is denoted by τ_0_, and the quencher concentration is denoted by [Q]^62^.

**Figure 8:**
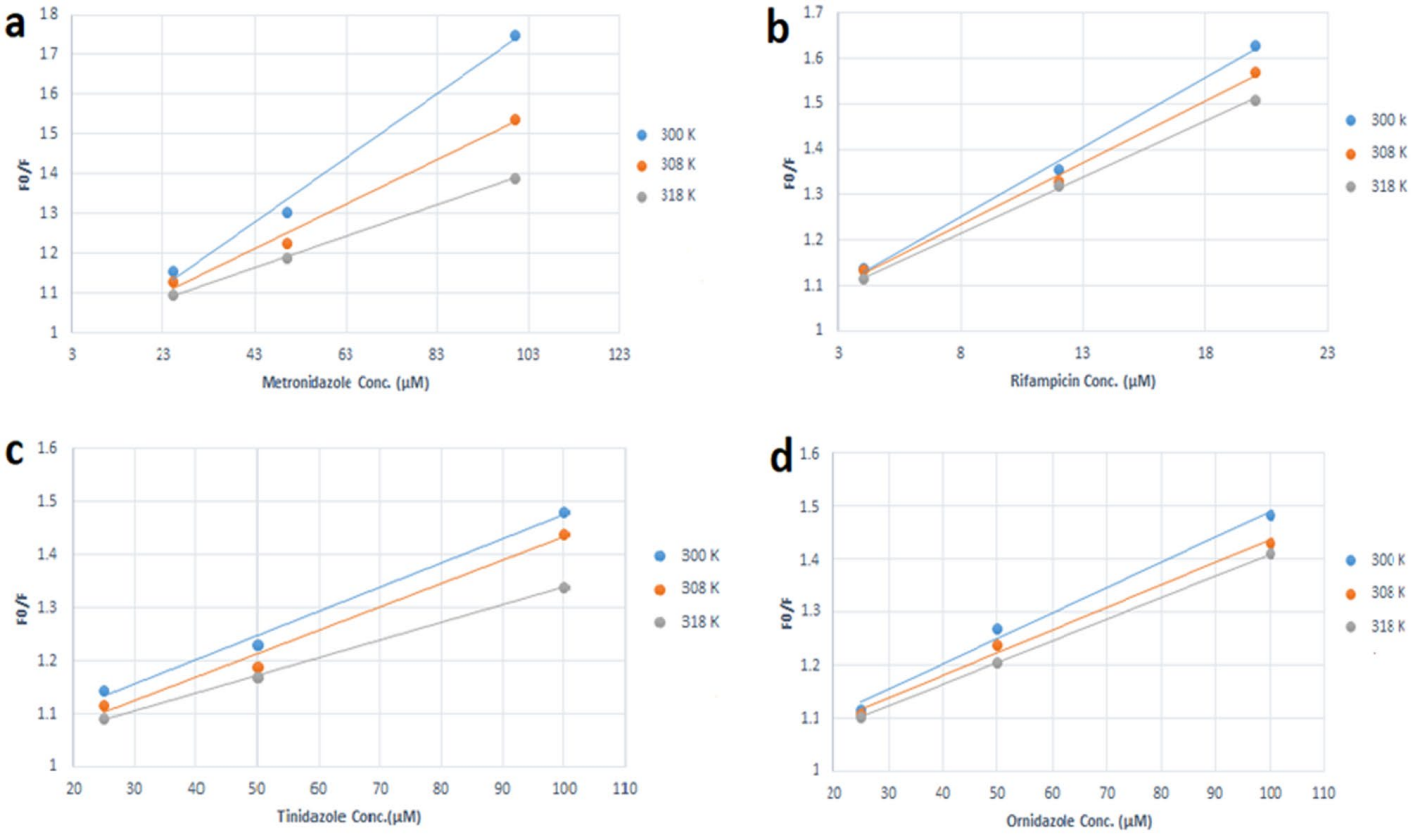
Stern–Volmer plots between F_0_/F and concentration (μM) of MNZ (**a**), RFP (**b**), TNZ (**c**), and ONZ (**d**).

The static quenching mechanism was proved using the Stern–Volmer plots as the values of K_sv_ decreased when temperature increased (300, 308, 318 K), as shown in Fig. 8. As a result, it was concluded that, both static quenching mechanism and inner filter effect were found to be responsible for the SN-CQDs’ fluorescence intensity quenching in presence of the studied drugs.

### Optimization of experimental conditions

#### Effect of pH

**Figure 9:**
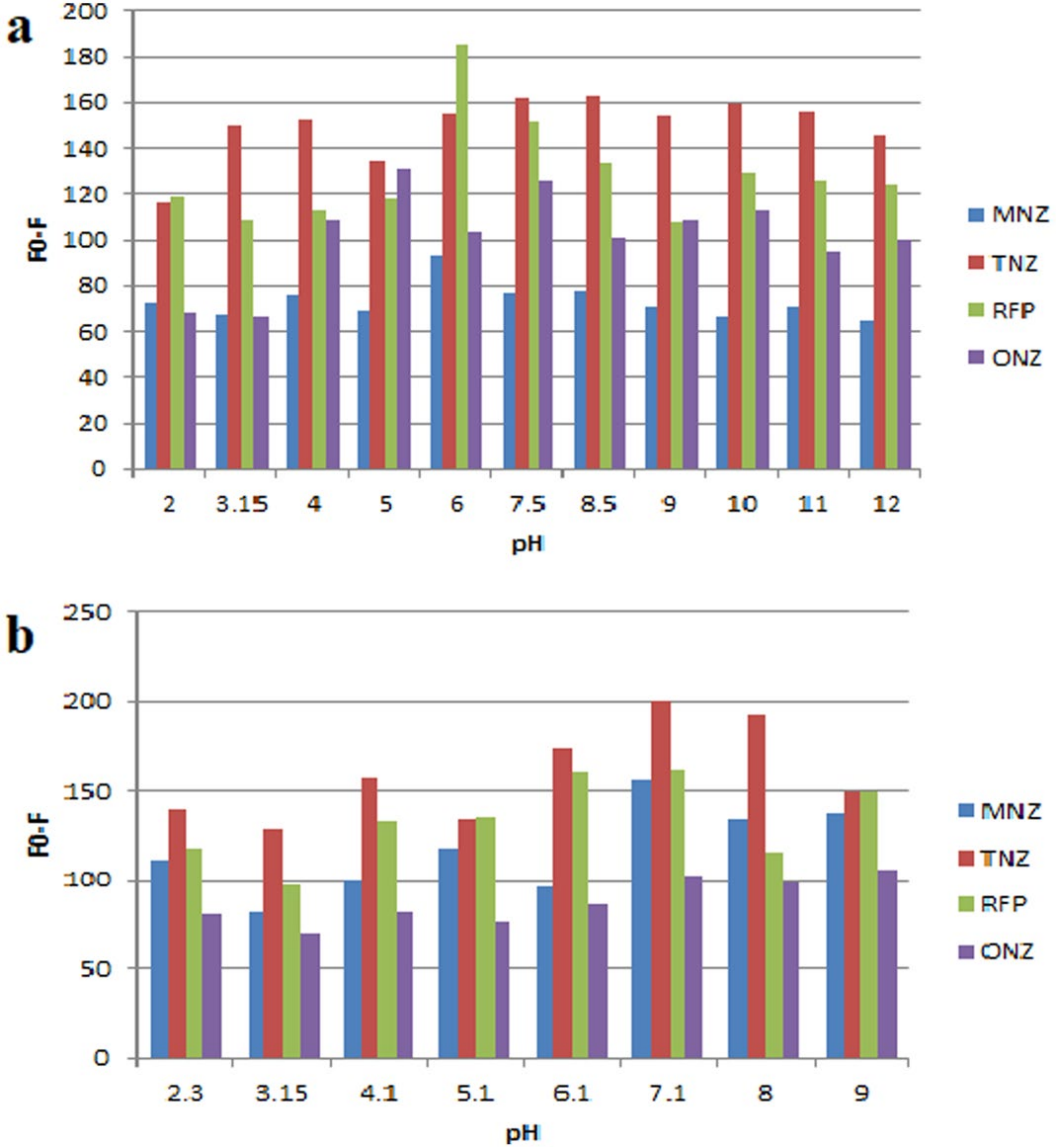
(**a**) Effect of Britton-Robinson buffer (pH 2–12) on fluorescence quenching of SN-CQDs by MNZ (50.0 μM), ONZ (50.0 μM), RFP (10.0 μM), and TNZ (50.0 μM), (**b**) Effect of phosphate buffer (pH 2–9) on fluorescence quenching of SN-CQDs by MNZ (50.0 μM), ONZ (50.0 μM), RFP (10.0 μM), and TNZ (50.0 μM).

The influence of pH on the quenching of SN-CQDs fluorescence intensity by the studied drugs was studied utilizing various buffer solutions including, phosphate buffer and Britton–Robinson buffer covering pH range of 3–9.5 and 2.1–12, respectively. It was found that, phosphate buffer (0.05 M) with pH of 7.1 produced the highest fluorescence quenching for both TNZ and MNZ, and Britton–Robinson (0.02) with pH of 5.1 and 6.0 produced the highest fluorescence quenching for ONZ and RFP, respectively (Fig. 9). Different volumes of the selected buffers in the range of 0.5–3.0 mL were studied. It was found that 1.0 mL of the buffer was sufficient to produce the maximum fluorescence quenching for MNZ, ONZ and RFP and 0.5 mL was the optimum volume for TNZ.

#### Effect of incubation time

**Figure 10:**
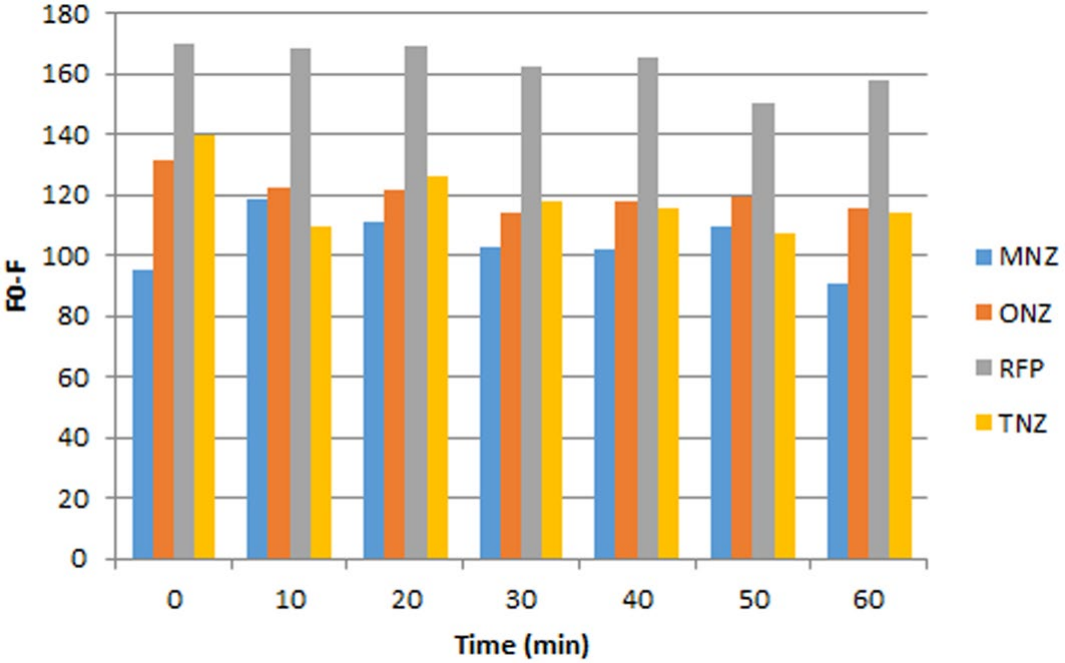
Effect of incubation time on fluorescence quenching of SN-CQDs by MNZ (50.0 μM), ONZ (50.0 μM), RFP (10.0 μM), and TNZ (50.0 μM).

The influence of incubation time on the quenching of SN-CQDs fluorescence was also investigated. It was investigated after adding drugs to SN-CQDs at various time intervals ranging from 1.0 to 60.0 min. It was found that, the quenching of fluorescence intensity of SN-CQDs reached its maximum value after 10.0 min with MNZ, while it was immediately with TNZ, ONZ, and RFP, as shown in Fig. 10.

### Method validation

The suggested method was validated according to the International Conference of Harmonization (ICH) Q2 (R1) recommendations^63^.

#### Linearity and range

Plotting the concentration of the studied drugs in μM against the fluorescence quenching of SN-CODs was performed to obtain the calibration curves. As shown in Table 2, the studied drugs' linearity was obtained within the concentration ranges with ideal correlation coefficient of 0.9999. Linear regression can be expressed using Eqs. (5–8) below:

5 $$\left( {{\text{F}}_{0} - {\text{F}}} \right) = 15.899{\text{C}} + 55.886\quad \left( {{\text{r}} = 0.9999} \right)\;{\text{for RFP}}$$

6 $$\left( {{\text{F}}_{0} - {\text{F}}} \right) = 2.2406{\text{C}} + 32.813\quad \left( {{\text{r}} = 0.9999} \right)\;{\text{for TNZ}}$$

7 $$\left( {{\text{F}}_{0} - {\text{F}}} \right) = 2.667{\text{C}} + 2.8357\quad \left( {{\text{r}} = 0.9999} \right)\;{\text{for ONZ}}$$

8 $$\left( {{\text{F}}_{0} - {\text{F}}} \right) = 2.67{\text{C}} + 47.468\quad \left( {{\text{r}} = 0.9999} \right)\;{\text{for MNZ}}$$

where F_0_ is the SN-CQDs’ native fluorescence intensity, F is the SN-CQDs fluorescence intensity in presence of drugs, C represents the drugs concentration in μM, and r refers to the correlation coefficient.

**Table 2:** Analytical performance data of the proposed method.

Parameters	RFP	ONZ	TNZ	MNZ
Linearity range (µM)	1.0–30.0	6.0–200.0	10.0–200.0	5.0–100.0
Limit of detection, LOD (µM)	0.31	0.57	1.76	0.75
Limit of quantitation, LOQ (µM)	0.93	1.74	5.32	2.28
Slope (b)	15.89	2.67	2.24	2.67
Intercept (a)	55.89	2.84	32.81	47.47
Correlation coefficient (r)	0.9999	0.9999	0.9999	0.9999
S.D. of the residuals, S_y/x_	2.45	1.63	1.78	1.33
S.D. of the intercept, S_a_	1.48	0.46	1.19	0.61
S.D. of the slope, S_b_	0.095	0.0098	0.0106	0.0157
Percentage relative standard deviation, % RSD	1.95	1.14	1.97	1.60
Percentage relative error, % Error	0.80	0.47	0.69	0.60

#### LOD and LOQ

As shown in Table 2, the obtained LOD and LOQ values demonstrate that, the suggested method is sensitive enough to determine the analytes in dosage forms and biological fluids. The following Eqs. (9, 10) were used to calculate LOD and LOQ values^64^:

9 $${\text{LOD}} = 3.3\,{{{\text{S}}_{{\text{a}}} } \mathord{\left/ {\vphantom {{{\text{S}}_{{\text{a}}} } {\text{b}}}} \right. \kern-0pt} {\text{b}}}$$

10 $${\text{LOQ}} = 10\,{{{\text{S}}_{{\text{a}}} } \mathord{\left/ {\vphantom {{{\text{S}}_{{\text{a}}} } {\text{b}}}} \right. \kern-0pt} {\text{b}}}$$

where S_a_ refers to the standard deviation of a regression line’s intercept, and b refers to the linear calibration curve’s slope.

#### Accuracy and precision

By comparing the assay results for the cited drugs to those obtained by the reported methods^12–14,65^, the developed method’s accuracy and precision were investigated. The results in Table 3 show that, there were no significant differences in precision and accuracy between the proposed and comparison methods. The Variance ratio F-test and the Student t-test were used to statistically analyze the data, respectively^64^. Moreover, the method's precision was evaluated by studying intra-day and inter-day precisions. They were estimated using three different concentrations, each with three replicates on the same day and on three consecutive days, respectively. Values of % RSD of less than 2% were obtained, demonstrating the suggested method’s acceptable precision (Table S1).

**Table 3:** Application of the proposed method for the determination of RFP, TNZ, ONZ, and MNZ in raw materials.

Parameter/compound	Proposed method		
	Conc. taken (μM)	Conc. found (μM)	% recovery^a^
RFP	1.0	1.013	101.32
	2.0	2.087	104.39
	5.0	5.034	100.68
	10.0	9.93	99.32
	20.0	19.76	98.84
	30.0	30.165	100.55
Mean			100.85
± SD			1.96
% RSD			1.945
% Error			0.801
	Comparison method^65^		
Mean ± SD	98.4 ± 0.53		
N	3		
Student t-test	2.06 (2.36)^b^		
Variance ratio F-value	13.75 (19.29)^b^		
TNZ	10.0	9.72	97.17
	25.0	24.72	98.87
	50.0	51.11	102.23
	100.0	99.09	99.10
	150.0	150.52	100.35
	200.0	199.83	99.91
Mean			99.61
± SD			1.69
% RSD			1.696
% Error			0.69
	Comparison method^14^		
Mean ± SD	99.0 ± 0.85		
N	3		
Student t-test	0.57 (2.36)^b^		
Variance ratio F-value	3.91 (19.29)^b^		
ONZ	6.0	6.05	100.83
	12.0	11.91	99.52
	25.0	25.41	101.63
	50.0	50.31	100.62
	100.0	98.89	98.89
	200.0	200.43	100.21
Mean			100.28
± SD			1.14
% RSD			1.14
% Error			0.467
	Comparison method^13^		
Mean ± SD	101.3 ± 0.88		
N	3		
Student t-test	1.53(2.36)^b^		
Variance ratio F-value	1.34(19.29)^b^		
MNZ	5.0	4.92	98.37
	10.0	9.95	99.52
	25.0	24.32	97.28
	40.0	40.61	101.53
	50.0	50.23	100.47
	75.0	75.59	100.78
	100.0	99.37	99.37
Mean			99.62
± SD			1.60
% RSD			1.602
% Error			0.604
	Comparison method^12^		
Mean ± SD	98.03 ± 1.20		
N	3		
Student t-test	1.64 (2.31)^b^		
Variance ratio F-value	1.47(19.33)^b^		

^a^Mean of three determinations.

^b^Values in parenthesis are the tabulated t- and F- values at p = 0.05^64^.

#### Robustness

The proposed method's robustness was evaluated by studying the influence of minor deliberate changes in the experimental conditions influencing fluorescence sensing, such as the volume of reagent, incubation period, buffer pH, and buffer volume. As illustrated in Table S2, the results revealed that, minor changes in experimental conditions had no significant changes on the fluorescence intensity quenching.

#### Selectivity

**Figure 11:**
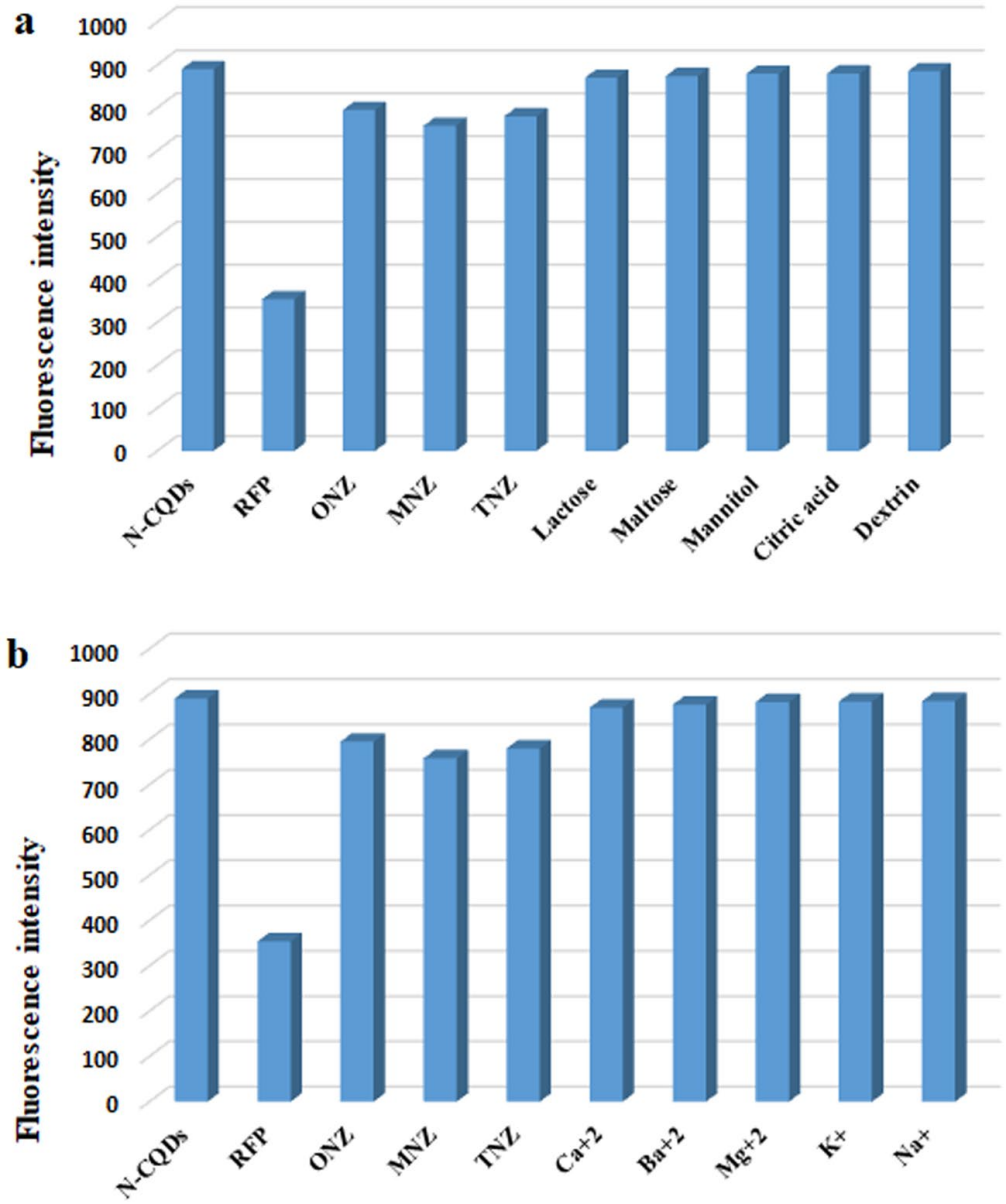
(a) The response of possible interfering excipients, (b) The selectivity of SN-CQDs towards ONZ, RFP, TNZ, and MNZ in presence of different metal ions.

The ability of the suggested method to detect RFP in presence of co-administered drugs like isoniazid without interference demonstrated the selectivity of the method (its tolerance limit was 40.0 μg/mL). It was also demonstrated by detecting interferences caused by common excipients and additives. It was found that, with high % recoveries (99.27–100.05) and low % RSD values (less than 2%), the suggested method could efficiently determine cited drugs in their dosage forms, demonstrating the developed method’s high selectivity (Table 4). The possible interfering excipients as lactose, maltose, mannitol, dextrin, and citric acid were studied in details and confirmed the high selectivity of the method, since they almost did not affect the fluorescence intensity of the SN-CQDs (Fig. 11a). Similarly, the method selectivity was proved, by its ability to detect the studied drugs in the presence of different metal ions like Na^+^, K^+^, Ca^+2^, Mg^+2^, and Ba^+2^ without any interference (Fig. 11b). Analyzing the examined drugs in spiked human plasma also verified the method selectivity. The suggested method demonstrated enough selectivity to determine the studied drugs in the complicated biological matrices with low % RSD values (less than 4.52%) and high mean percentage recoveries (99.44–100.29) for the studied drugs, with no interference from plasma endogenous components (Table 5).

**Table 4:** Application of the proposed method for the determination of the drugs in dosage forms.

Parameter	Proposed method			Comparison method^65^
	Conc.taken (μM)	Conc.found (μM)	% recovery^a^	% recovery^a^
RFP				
REP capsules (300 mg/capsule)	5.0	5.09	101.91	101.19
	10.0	9.89	98.87	97.9
	15.0	14.90	99.36	97.8
Mean			100.05	98.96
± SD			1.63	1.93
% RSD			1.628	1.949
Student t-test	0.74 (2.77)^b^			
Variance ratio F-value	1.39 (19.0)^b^			

Parameter	Proposed method			Comparison method^14^
	Conc.taken (μM)	Conc.found (μM)	% recovery^a^	% recovery^a^
TNZ				
Protozole® coated tablets (TNZ, 500 mg/tablet)	50.0	49.57	99.14	97.7
	100.0	101.67	101.67	97.39
	150.0	147.71	98.47	95.19
Mean			99.76	96.76
± SD			1.69	1.37
% RSD			1.691	1.412
Student t-test	2.39 (2.77)^b^			
Variance ratio F-value	1.52 (19.0)^b^			

Parameter	Proposed method			Comparison method^13^
	Conc.taken (μM)	Conc.found (μM)	% recovery^a^	% recovery^a^
ONZ				
Astranida® coated tablets (ONZ, 500 mg/tablet)	25.0	24.66	98.64	98.9
	50.0	48.64	97.80	101.14
	75.0	76.02	101.36	102.08
Mean			99.27	100.71
± SD			1.86	1.63
% RSD			1.875	1.623
Student t-test	1.01 (2.78)^b^			
Variance ratio F-value	1.29 (19.0)^b^			

Parameter	Proposed method			Comparison method^12^
	Conc.taken (μM)	Conc.found (μM)	% recovery^a^	% recovery^a^
MNZ				
Flagyl® coated tablets (MNZ, 500 mg/tablet)	10.0	9.95	99.48	98.2
	25.0	24.83	99.31	98.9
	40.0	39.81	99.53	99.1
Mean			99.44	98.73
± SD			0.12	0.47
% RSD			0.116	0.479
Student t-test	2.52 (2.78)^b^			
Variance ratio F-value	16.79 (19.0)^b^			

^a^Mean of three determinations.

^b^Values in parenthesis are the tabulated t- and F-values at p = 0.05^64^.

**Table 5:** Application of the proposed method for determination of the drugs in spiked human plasma samples.

Parameter	Conc. taken (μM)	Conc. found (μM)	% recovery^a^
RFP			
	1.0	1.01	101.34
	3.0	2.96	98.66
	4.0	4.03	100.67
Mean ± SD			100.22 ± 1.40
%RSD			1.392
TNZ			
	10.0	10.26	102.60
	20.0	19.48	97.40
	30.0	30.26	100.87
Mean ± SD			100.29 ± 2.65
%RSD			2.640
ONZ			
	10.0	9.63	96.26
	20.0	20.52	102.62
	45.0	44.85	99.67
Mean ± SD			99.44 ± 4.50
%RSD			4.522
MNZ			
	20.0	20.09	100.48
	30.0	29.71	99.05
	35.0	35.19	100.54
Mean ± SD			100.02 ± 0.84
%RSD			0.843

^a^Mean of three determinations.

### Method applications

#### Analysis of the drugs in dosage forms

The cited drugs' quantitative determination in their commercial dosage forms was accomplished effectively using the suggested method. The derived regression equations were used to calculate the concentrations of the investigated drugs. Table 4 shows that the average % recoveries (99.27–100.05) of different concentrations of the drugs in dosage forms were satisfactory. These results and those obtained by the comparison methods were in good agreement^12–14,65^, proving the established method's good accuracy and precision.

#### Determination of the drugs in human plasma

According to the data provided by the developed method, it possesses the sufficient selectivity and sensitivity to determine and/or detect the drugs in spiked human plasma samples (Fig. S2). The maximum plasma concentration (C_max_) was 10.54 mg/L for RFP and 35.7 mg/L after single oral dose of 2 g to 3 subjects for TNZ^66^. For MNZ, the C_max_ was reported to be from 4.5 to 11.6 (mean 6.9) mg/L after single oral dose of 400 mg every 8 h, and 28.9 and 18 mg/L for IV MNZ, and 500 mg every 8 h, respectively^66^. For ONZ, The C_max_ is 10.9 μg/mL reached after 2–4 h of administration of oral dose of 750 mg^67^. The obtained mean % recoveries of the 4 drugs in plasma samples were (99.44–100.29) with % RSD of (0.843–4.522) after data statistical analysis (Table 5). Linear regression can be expressed using the following Eqs. (11, 12, 13, 14):

11 $$\left( {{\text{F}}_{0} - {\text{F}}} \right) = 5.9451{\text{C}} - 83.98\quad \left( {{\text{r}} = 0.9995} \right)\;{\text{for MNZ}}$$

12 $$\left( {{\text{F}}_{0} - {\text{F}}} \right) = 30.765{\text{C}} - 0.2951\quad \left( {{\text{r}} = 0.9997} \right)\;{\text{for RFP}}$$

13 $$\left( {{\text{F}}_{0} - {\text{F}}} \right) = 5.407{\text{C}} - 9.6033\quad \left( {{\text{r}} = 0.9990} \right)\;{\text{for TNZ}}$$

14 $$\left( {{\text{F}}_{0} - {\text{F}}} \right) = 7.1522{\text{C}} - 3.5059\quad \left( {{\text{r}} = 0.9997} \right)\;{\text{for ONZ}}$$

## Conclusion

The current study describes a sensitive,-simple, and-rapid spectrofluorimetric approach for the determination of four pharmaceutically-important nitro compounds, including RFP, TNZ, ONZ, and MNZ in their dosage forms, and spiked human plasma samples. The suggested method relies on the use of SN-CQDs as fluorescent nanosensors for cited drugs determination. To synthesize SN-CQDs, citric acid was employed as a carbon source and thiosemicarbazide as nitrogen and sulfur source. Different approaches were used to characterize the prepared SN-CQDs. The prepared dots' quantum yield was also calculated. A novel spectrofluorimetric method for the determination of the studied drugs was developed depending on the quantitative fluorescence quenching of SN-CQDs that occured as a result of the increased concentrations of the investigated drugs. The-mechanism of quenching was studied and discussed. The suggested method was validated in accordance with ICHQ2 (R1) recommendations.

## Supplementary Information

Supplementary Information.

## Data Availability

The datasets generated and/or analyzed during the current study are available from the corresponding author upon reasonable request.
