# Supplementary material for: Sulfur and nitrogen co-doped carbon quantum dots as fluorescent probes for the determination of some pharmaceutically-important nitro compounds
Source: Sci Rep. 2023 Apr 4;13:5502. doi: 10.1038/s41598-023-32494-8 (PMC10073125; doi:10.1038/s41598-023-32494-8)
Supplement: Supplementary file 1 — Supplementary Information. [file 41598_2023_32494_MOESM1_ESM.pdf]

Supplementary Material

Sulfur and nitrogen co-doped carbon quantum dots as fluorescent probes for the determination of some pharmaceutically-important nitro compounds

Galal Magdy[a*], Shaimaa Ebrahim[a], Fathalla Belal[b], Ramadan A. El-Domany[c], Ahmed M. Abdel-Megied[a,d]

[a] Pharmaceutical Analytical Chemistry Department, Faculty of Pharmacy, Kafrelsheikh University, Kafrelsheikh, P.O. Box 33511, Egypt.

[b] Pharmaceutical Analytical Chemistry Department, Faculty of Pharmacy, Mansoura University, Mansoura, P.O. Box 35516, Egypt.

[c] Microbiology and Immunology Department, Faculty of Pharmacy, Kafrelsheikh University, Kafrelsheikh, P.O. Box 33511, Egypt.

[d] Department of Pharmaceutical Sciences, Notre Dame of Maryland University, School of Pharmacy, Baltimore, MD 21210, USA.

***Corresponding author:** Galal Magdy

E-mail address: galal_magdy@pharm.kfs.edu.eg

Supplementary Figures:

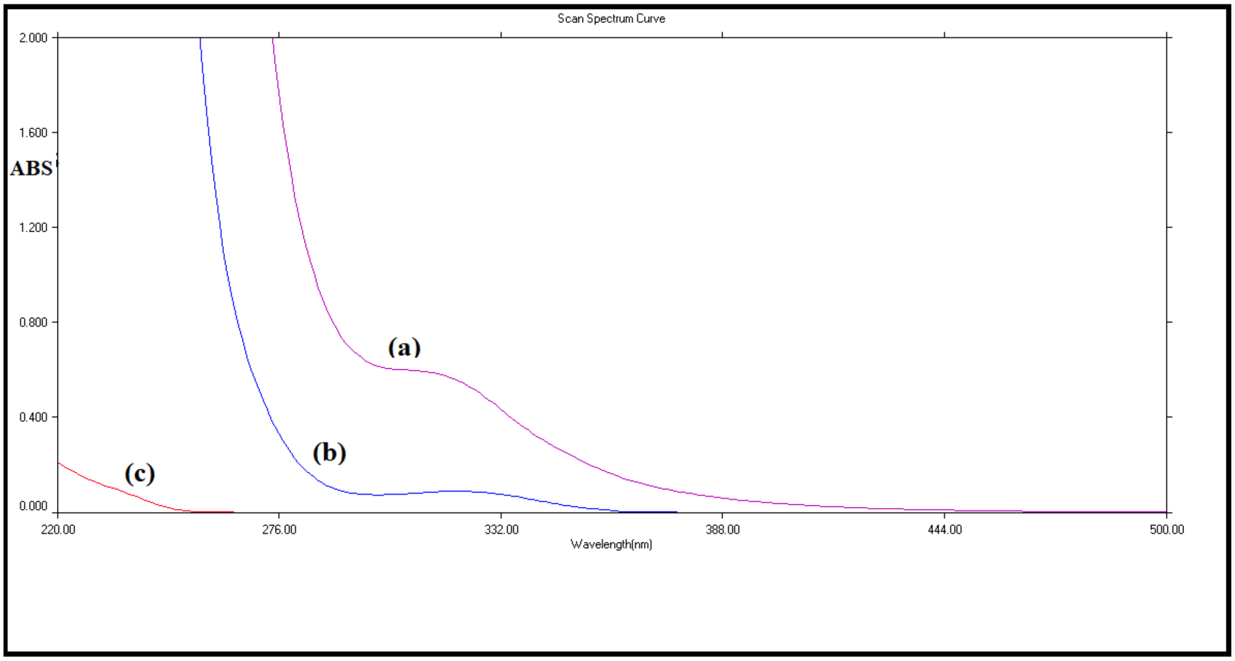

Fig. S1 UV-Visible absorption spectra of SN-CQDS (a), TSC (b), and CA (c).

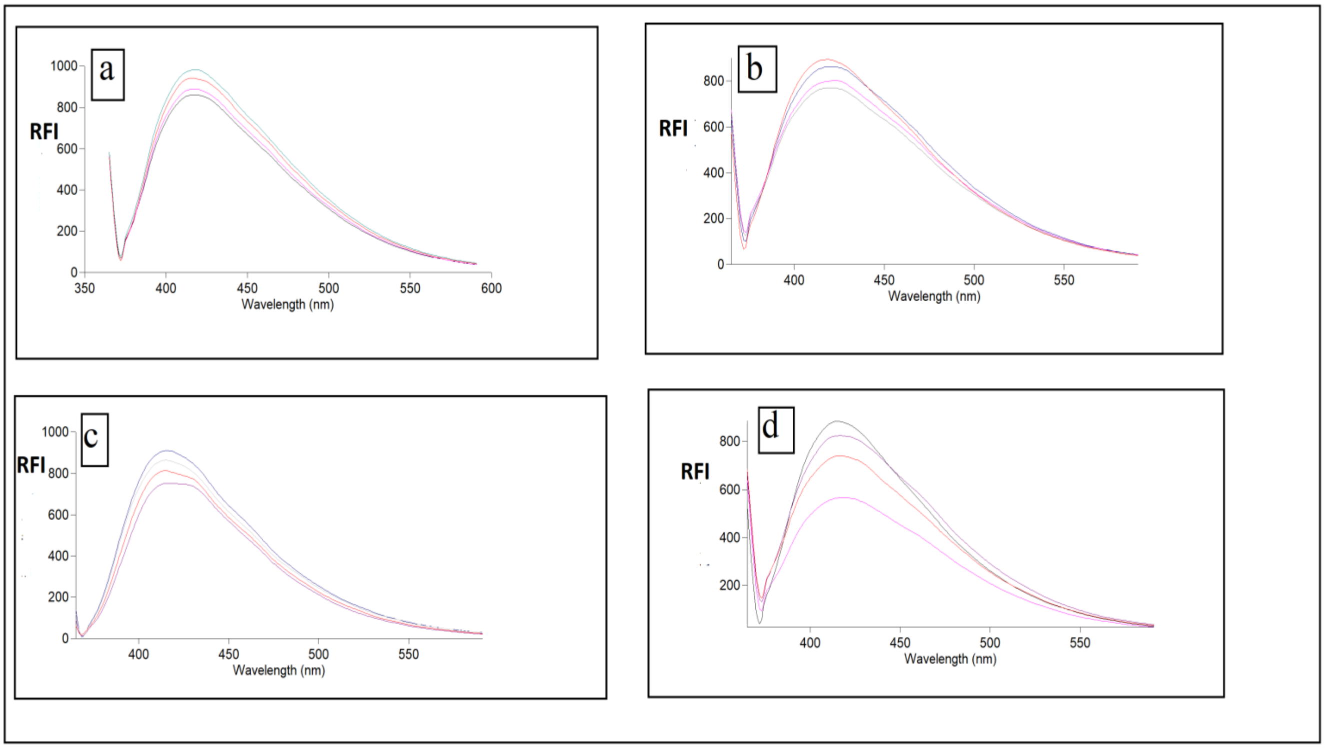

Fig. S2 Fluorescence emission spectra of SN-CQDs in spiked human plasma upon addition of various concentrations of (a) MNZ (from top to bottom: 0, 20.0, 30.0, 35.0), (b) RFP (from top to bottom: 0, 1.0, 3.0, 4.0), (c) TNZ (from top to bottom: 0, 10.0, 20.0, 30.0), and (d) ONZ (from top to bottom: 0, 10.0, 20.0, 45.0).

Supplementary Tables:

Table S1: Intra-day and inter-day precision data for the determination of RFP, ONZ, TNZ and MNZ by the proposed method

Analyte	Conc.taken (μM)	Intra-day[a]			Inter-day[b]		
		Conc.found ± S.D. (μM)	%RSD	%Error[c]	Conc.found ± S.D. (μM)	%RSD	%Error[c]
**RFP**	**4.0**	3.95±0.90	0.91	0.53	3.99±1.85	1.85	1.07
	**12.0**	11.94±0.47	0.47	0.27	11.96±0.31	0.31	0.18
	**20.0**	19.75±0.24	0.25	0.14	19.81±0.04	0.04	0.02
**ONZ**	**12.0**	12.06±1.42	1.41	0.82	11.85±0.49	0.49	0.28
	**50.0**	50.29±0.15	0.15	0.08	50.45±0.54	0.53	0.31
	**100.0**	99.60±0.62	0.62	0.36	99.64±1.25	1.25	0.72
**TNZ**	**25.0**	24.57±0.85	0.86	0.50	24.623±0.88	0.89	0.51
	**50.0**	51.31±0.54	0.52	0.30	51.044±0.29	0.28	0.16
	**150.0**	150.92±0.27	0.26	0.15	150.76±0.14	0.14	0.08
**MNZ**	**10.0**	9.91±0.44	0.44	0.25	9.95±0.81	0.81	0.47
	**40.0**	40.36±0.64	0.63	0.37	40.53±0.17	0.17	0.10
	**75.0**	75.69±0.12	0.12	0.07	75.59±0.16	0.16	0.09

Each result is the average of three separate determinations.

[a] Within the day.

[b] Three consecutive days.

[c] % Error = % RSD/ √n.

Table S2: Robustness evaluation of the proposed method

Variation	RFP	
1- Volume of SN-CQDs (100.0 µL ± 1)	% Recovery	%RSD
99.0 µL	101.08	1.10
100.0 µL	100.91	0.46
101.0 µL	101.28	0.38
2- Britton-Robinson buffer pH (6.0 ±0.2)	% Recovery	%RSD
pH=6.2	101.00	0.84
pH=6.0	100.92	0.47
pH=5.8	100.83	1.18
3- Volume of buffer (1000 µL ± 5.0)	% Recovery	%RSD
995 µL	100.07	0.53
1000 µL	100.92	0.47
1005 µL	100.39	0.17
**Variation**	**TNZ**	
1- Volume of SN-CQDs (100.0 µL ± 5)	% Recovery	%RSD
95.0 Ml	97.93	1.173
100.0 µL	100.51	1.031
105.0 µL	97.52	1.42
2- Phosphate buffer pH (7.1 ± 0.2)	% Recovery	%RSD
pH=6.9	98.62	1.61
pH=7.1	100.51	1.031
pH=7.3	101.75	1.17

3- Volume of phosphate buffer (500 µL ± 5)	% Recovery	%RSD
495 µL	100.7	1.53
500 µL	100.51	1.031
505 µL	98.22	1.8
Variation	ONZ	
1- Volume of SN-CQDs (100.0 µL ± 1)	% Recovery	%RSD
99.0 µL	99.59	0.77
100.0 µL	101.81	0.62
101.0 µL	100.08	1.64
2- Britton-Robinson buffer pH (5.1 ±0.2)	% Recovery	%RSD
pH=4.9	101.18	1.88
pH=5.1	101.81	0.62
pH=5.3	99.68	0.88
3- Volume of Britton-Robinson buffer (1000 µL ± 5.0)	% Recovery	%RSD
995 µL	98.52	1.99
1000 µL	101.81	0.62
1005 µL	99.30	1.08
Variation	MNZ	
1- Volume of SN-CQDs (100.0 µL ± 1)	% Recovery	%RSD
99.0 µL	100.58	0.38
100.0 µL	101.25	0.46
101.0 µL	100.88	0.32

2- Incubation time (10 min ± 1 min.)	% Recovery	%RSD
9 min	100.87	0.48
10 min	101.25	0.46
11 min	100.58	0.49
3- Phosphate buffer pH (7.1 ± 0.2)	% Recovery	%RSD
pH=6.9	100.84	0.34
pH=7.1	101.25	0.46
pH=7.3	100.38	0.58
4- Volume of phosphate buffer (1000 µL ±5.0)	% Recovery	%RSD
995 µL	101.31	0.39
1000 µL	101.25	0.46
1005 µL	100.71	0.77
